# The Association Between Hypertension in Pregnancy and Preterm Birth with Fetal Growth Restriction in Singleton and Twin Pregnancy: Use of Twin Versus Singleton Charts

**DOI:** 10.3390/jcm9082518

**Published:** 2020-08-05

**Authors:** Erkan Kalafat, Aisha Abiola, Basky Thilaganathan, Amar Bhide, Asma Khalil

**Affiliations:** 1 Fetal Medicine Unit, St George’s Hospital, St George’s University of London, Cranmer Terrace, London SW17 0RE, UK; mail@erkankalafat.com (E.K.); m1700176@sgul.ac.uk (A.A.); basky@pobox.com (B.T.); abhide@sgul.ac.uk (A.B.); 2 Department of Statistics, Faculty of Arts and Science, Middle East Technical University, 06800 Ankara, Turkey; 3 Department of Obstetrics and Gynecology, Faculty of Medicine, Ankara University, 06230 Ankara, Turkey; 4 Vascular Biology Research Centre, Molecular and Clinical Sciences Research Institute, St George′s University of London, Cranmer Terrace, London SW17 0RE, UK; 5 Twins Trust Centre for Research and Clinical excellence, St George’s Hospital, Blackshaw road, Tooting, London SW17 0QT, UK

**Keywords:** multiple, growth, customized, adverse outcome, preeclampsia, gestational hypertension

## Abstract

Objective: To compare the rates of fetal growth restriction (FGR) in singleton and twin pregnancies using singleton and twin-specific birthweight standards. Methods: The study included liveborn twin and singleton pregnancies between January 2000 and January 2019. Hypertensive disorders of pregnancy (HDP) included gestational hypertension and pre-eclampsia. The study outcomes were FGR or small-for-gestational-age (SGA) at birth as assessed using singleton and twin reference charts. Results: The analysis included 1473 twin and 62,432 singleton pregnancies. In singleton pregnancies the risk of PTB <34 weeks without HDP (OR 2.82, *p* < 0.001), delivery ≥34 weeks with HDP (OR 2.38, *p* < 0.001), and PTB <34 weeks with HDP (OR 13.65, *p* < 0.001) were significantly higher in the pregnancies complicated by FGR compared to those without. When selective fetal growth restriction (sFGR) was assessed using the singleton standard, the risk of PTB <34 weeks without HDP (OR 1.03, *p* = 0.872), delivery ≥34 weeks with HDP (OR 1.36, *p* = 0.160) were similar in the pregnancies complicated by sFGR compared to those without, while the risk of PTB <34 weeks with HDP (OR 2.41, *p* = 0.025) was significantly higher in the pregnancies complicated by sFGR compared to those without. When sFGR was assessed using the twin-specific chart, the risk of PTB <34 weeks without HDP (OR 3.55, *p* < 0.001), delivery ≥34 weeks with HDP (OR 3.17, *p* = 0.004), and PTB <34 weeks with HDP (OR 5.69, *p* < 0.001) were significantly higher in the pregnancies complicated by sFGR compared to those without. The stronger and more consistent association persisted in the subgroup analyses according to chorionicity. The strength of association in dichorionic twin pregnancies resembles that of the singletons more closely and consistently when the FGR was diagnosed using the twin-specific charts. Conclusion: FGR in twin pregnancies has a stronger and more consistent association with HDP and PTB when using twin-specific rather than singleton charts. This study provides further evidence supporting the use of twin-specific charts when assessing fetal growth in twin pregnancies.

## 1. Introduction

Twin pregnancies constitute 1.6% of all births but they contribute disproportionately to the perinatal mortality and morbidity by representing 20% of preterm births and 5.9% of stillbirths [1]. Empirical evidence suggests that the implementation of national guidelines, based on twin-specific research, could reduce stillbirth and neonatal death in twin pregnancies [2,3]. Nevertheless, stillbirth and neonatal death are still more common in twin compared to singleton pregnancies [4]. Therefore, more research efforts are needed first to understand the reasons, second to identify potential interventions to reduce the risk of perinatal mortality and morbidity, and third to identify the challenges to implement these interventions.

Preterm birth (PTB), growth disorders and monochorionicity-related complications are among the most important contributors to the excess perinatal mortality and morbidity in twin pregnancies. Furthermore, growth assessment in twins has been identified by the Global Twins and Multiples Priority Setting Partnership as one of the 10 most important research priorities for the future health of multiples and their families [5]. Twin-specific growth and birthweight charts are available but their use in clinical practice is currently controversial [6,7,8]. Most studies use singleton standards for the evaluation of twins [9,10], but the results of these studies may change significantly if twin-specific standards are used [11]. Moreover, the diagnostic criteria for selective fetal growth restriction (sFGR) do not recommend a specific reference standard to use, despite including weight percentile thresholds as part of the diagnostic criteria [12,13]. An evidence-based consensus on the management of growth restriction in twins is unlikely to be achievable before the harmonization of which reference standards to use while assessing the fetal growth or birthweight [14,15,16,17].

Recent evidence has demonstrated that the use of twin-specific charts better identifies the pregnancies at increased risk of adverse perinatal outcome [18,19]. However, there is a general reluctance to take up their use into routine clinical practice, as the use of twin charts is still considered controversial. This study aims to compare the association of FGR with hypertensive disorders in pregnancy (HDP) and PTB in singleton and twin pregnancies using singleton versus twin-specific birthweight standards.

## 2. Experimental Section

### 2.1. Study Population

This was a cohort study with retrospective analysis of prospectively collected data of unselected liveborn twin and singleton pregnancies at St George’s Hospital between January 2000 and January 2019. Pregnancies were identified by searching the electronic maternity records (ViewPoint version 5.6.26.148, ViewPoint Bildverarbeitung GMBH, Wessling, Germany). The exclusion criteria included major fetal structural anomalies or aneuploidy; twin-to-twin transfusion syndrome (TTTS); twin reversed arterial perfusion sequence (TRAPS); twin anemia–polycythemia sequence (TAPS); missing gestational age at delivery, birthweight or HDP data; and women with pre-pregnancy hypertension. Pregnancy outcomes were ascertained from the maternity database and neonatal records.

### 2.2. Study Variables and Outcomes

The chorionicity was determined based on the number of placentas and the presence or absence of the lambda sign at the intertwin membrane–placenta junction, as well as the inter-twin membrane thickness at the site of its insertion in the chorion at 11–14 weeks, or the number of placentas and the fetal gender after 14 weeks’ gestation [19,20]. Gestational age (GA) was determined in the first trimester according to the crown-rump length of the fetus (singleton pregnancies) or the larger fetus (twin pregnancies) in cases of spontaneous conception and according to the time of in vitro fertilization for pregnancies conceived via assisted reproductive technology [21]. After 14 weeks’ gestation, GA was determined using the head circumference of the fetus (singleton pregnancies) or the larger fetus (twin pregnancies) in cases of spontaneous conception and according to the time of in vitro fertilization for pregnancies conceived via assisted reproductive technology [19,22].

Small-for-gestational-age (SGA) was defined as birthweight centile below the tenth and FGR in singleton was defined as weight centile below the third. Although the recent Delphi consensus statement did not propose a definition for sFGR at birth, we applied the antenatal criteria of one twin below the tenth weight centile and a weight discordance of more than 25% or the solitary criteria of one twin with birthweight below the third centile [13]. The weight centiles were assessed using the singleton standard reported by Poon et al. and twin chorionicity-specific standards reported by Ananth et al. [7,23].

HDP included gestational hypertension and pre-eclampsia, as defined by the International Society of Hypertension in Pregnancy (ISSHP) guideline [24]. Women with new-onset hypertension (≥140 mm/Hg systolic or ≥90 mm/Hg diastolic on two separate occasions 12 h apart) were classified as gestational hypertension or pre-eclampsia according to the presence of systemic involvement (significant proteinuria or maternal organ dysfunction). Significant proteinuria was defined as a protein/creatinine ratio (PCR) ≥30 mg/mmol. Other criteria of systemic involvement included liver dysfunction (aspartate aminotransferase and alanine aminotransferase concentrations more than twice the upper reference range), reduced platelet count (<100,000/μL), hemolysis (increased lactate dehydrogenase concentration more than twice the upper reference range), renal failure (creatinine >90 umol/L), or neurological symptoms (persistent severe headache, seizures).

PTB included both spontaneous onset and iatrogenic (medically indicated) deliveries prior to 34 weeks’ gestation. The cut-off was chosen to be 34 weeks instead of traditional 37 weeks because most twin pregnancies are delivered between 37 to 38 weeks in our study center.

### 2.3. Statistical Analysis

Continuous variables were presented as median with interquartile ranges (IQR), and categorical variables were presented as number and percentage of the total. Logistic regression analyses were used to assess the association of HDP and GA at delivery with sFGR or SGA neonate. The receiver-operating characteristics curves were used and the areas under the curve (AUC) were compared using De Long’s test. *p* values below 0.05 were considered statistically significant. The statistical analysis was performed using RStudio (Version 1.0.136, Rstudio, Inc., Boston, MA, USA) statistical software.

## 3. Results

### 3.1. Study Population

The analysis included 1473 twin and 62,432 singleton pregnancies. There were 1177 (80.0%) dichorionic and 296 (20.0%) monochorionic twin pregnancies in the cohort. The incidence of HDP was 10.3% in twin and 5.0% in singleton pregnancies. Table 1 outlines baseline characteristics of the study population for singletons and twins. Twin pregnancies delivered at a significantly earlier gestational age compared to singletons (median: 36.9 vs. 40.0 weeks, *p* < 0.001). Preeclampsia (7.5% vs. 2.5%, *p* < 0.001) and SGA at birth (50.0% vs. 13.6%, *p* < 0.001) were significantly more common in twin than singleton pregnancies. The incidence of SGA newborn (24.8% vs. 50.0%, *p* < 0.001, McNemar’s chi-squared test) and sFGR at birth (22.0% vs. 11.3%, *p* < 0.001, McNemar’s chi-squared test) were significantly higher using the singleton compared to twin standards.

### 3.2. SGA and FGR in Singleton Pregnancies

The incidence of SGA and FGR in singleton pregnancies was 13.6% (*n* = 8470) and 3.9% (*n* = 2763), respectively. The risk of PTB <34 weeks without HDP (OR 2.82, *p* < 0.001), delivery ≥34 weeks with HDP (OR 2.38, *p* < 0.001), and PTB <34 weeks with HDP (OR 13.65, *p* < 0.001) were significantly higher in the pregnancies complicated by FGR compared to those without. The risk of PTB <34 weeks without HDP (OR 1.43, *p* < 0.001), delivery ≥34 weeks with HDP (OR 1.77, *p* < 0.001), and PTB <34 weeks with HDP (OR 10.26, *p* < 0.001) were significantly higher in the pregnancies complicated by SGA compared to those without (Table 2).

### 3.3. SGA and FGR in Twin Pregnancies

#### 3.3.1. FGF1. Selective FGR

When sFGR was assessed using the twin standard, the risk of PTB <34 weeks without HDP (OR 3.55, *p* < 0.001), delivery ≥34 weeks with HDP (OR 3.17, *p* = 0.004), and PTB <34 weeks with HDP (OR 5.69, *p* < 0.001) were significantly higher in the pregnancies complicated by sFGR compared to those without. When sFGR was assessed using the singleton standard, the risk of PTB <34 weeks without HDP (OR 1.03, *p* = 0.872) and delivery ≥34 weeks with HDP (OR 1.36, *p* = 0.160) were similar in the pregnancies complicated by sFGR compared to those without, while the risk of PTB <34 weeks with HDP was significantly higher in the pregnancies complicated by sFGR compared to those without (OR 2.41, *p* = 0.025) (Table 2).

The stronger and more consistent association using the twin reference charts persisted in the subgroup analyses according to chorionicity (Table 3). The AUC values of the models were also consistently higher when the sFGR outcome was assessed using the twin standard with the largest difference in the monochorionic twins subgroup (*p* < 0.050 for all) (Table 4, Figure S1).

#### 3.3.2. SGA of One or Both Twins

When SGA was assessed using the twin standard, the risk of PTB <34 weeks without HDP (OR 3.10, *p* < 0.001), delivery ≥34 weeks with HDP (OR 2.12, *p* < 0.001) and <34 weeks with HDP (OR 10.73, *p* < 0.001) were significantly higher in the pregnancies complicated by SGA compared to those without. When SGA was assessed using the singleton standard, the risk of PTB <34 weeks without HDP (OR 0.56, *p* < 0.001) was significantly lower, while the risk of PTB <34 weeks with HDP (OR 2.38, *p* = 0.040) was significantly higher in the pregnancies complicated by SGA compared to those without (Table 2). The risk of delivery ≥34 weeks with HDP (OR 1.41, *p* = 0.074) was not significantly difference in the pregnancies complicated by SGA compared to those without (Table 2).

The stronger and more consistent association using the twin reference charts persisted in the subgroup analysis according to chorionicity (Table 3). The AUC value of the models were also consistently higher (*p* < 0.050 for all) when any SGA outcome was assessed using the twin standard (Table 4, Figure S1) with the largest difference in the monochorionic twins’ subgroup.

**Figure 1:**
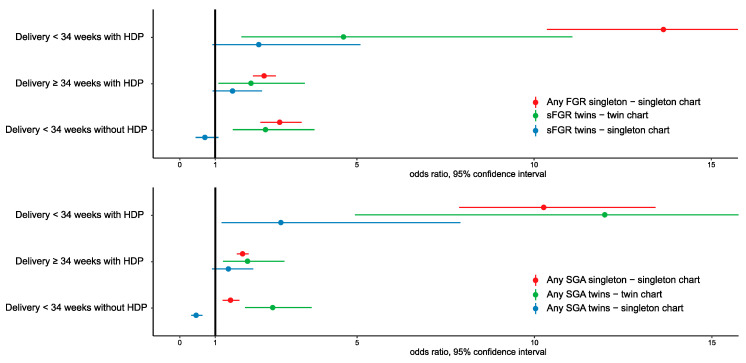
The association of hypertensive disorders of pregnancy (HDP) and preterm birth (PTB) with fetal growth restriction (FGR) or small-for-gestational age (SGA) in dichorionic twin versus singleton pregnancies. The association in twins resembles the association in singletons only when the outcome is assessed with twin-specific birthweight charts. SGA: small for gestational age–birth weight <10th centile, FGR: fetal growth restriction–birth weight <3rd centile, sFGR: selective fetal growth restriction–Delphi consensus, HDP: hypertensive disorders of pregnancy. The dot points represent the odds ratio and the lines represent the 95% confidence interval. Confidence intervals crossing the reference (black line, odds ratio = 1) indicate a statistically insignificant association. The x-line is truncated at 15.

The strength of association in dichorionic twin pregnancies resembles that of the singletons more closely and consistently when the FGR/SGA was assessed using the twin-specific charts (Figure 1).

## 4. Discussion

### 4.1. Summary of Main Results

Our study findings demonstrate that the association between FGR in twin pregnancies is significantly and consistently associated with HDP and PTB when using the twin-specific charts, but not with singleton charts. This association is valid in both dichorionic, as well as monochorionic twin pregnancies. This study adds to the body of evidence suggesting that singleton standards inflate the incidence of growth abnormalities in twins without identifying more pregnancies at risk of adverse outcomes.

### 4.2. Strengths and Limitations

There are several strengths to our study. Firstly, this was a large cohort of twin pregnancies and included both dichorionic and monochorionic pregnancies. Secondly, we excluded monochorionicity-specific complications, such as TTTS, TAPS and TRAPS and major fetal structural abnormalities in view of their know association with FGR and PTB. Thirdly, our analysis included a priori sensitivity analysis according to chorionicity. Fourthly, we decided to use singleton and twin standards which were not in clinical use in our center in order to minimize the risk of potential bias. Finally, we included livebirth pregnancies only in order to avoid the risk of overestimating the incidence of SGA or FGR; stillbirth is known to be associated with HDP and PTB. However, some limitations apply to the interpretation of our results. First, as we excluded stillbirth cases, this may have reduced the number of FGR/SGA cases. However, since we assessed the outcomes using the birthweight, the inclusion of stillbirth cases was not possible and might have introduced a systematic bias. Second, we did not incorporate some antenatal markers known to be associated with adverse outcomes such as estimated fetal weight or fetal Doppler [25]. However, we tested the association of the same factors by only changing the outcome variable. Therefore, whatever residual confounder effect left unaddressed should apply to all models in equal measure. Third, we only used one twin-specific birthweight standard among many available charts and the utility of other twin-specific standards is yet to be investigated. The same issue applies to singleton charts as well. Although there are more popular singleton charts available, we opted to use a chart that was not in active use, derived from a similar population in London and vetted for accuracy in our previous studies [8,26]. Lastly, we did not exclude spontaneous PTB without adverse events, which is a potential confounder in our analysis.

### 4.3. Interpretation of Study Findings and Comparison with Published Literature

Few studies looked at the association of twin-specific growth standards with clinical adverse outcomes [8,18]. The findings of the study by Kalafat et al. have suggested that twin-specific growth standards could identify the same number of twin pregnancies with SGA and stillbirth as the singleton charts, while labeling fewer pregnancies with liveborn fetuses as complicated with growth abnormalities. More recently, Proctor et al. have demonstrated that the association of HDP with live-born FGR babies is significantly stronger and more consistent when FGR is diagnosed using twin-specific charts. Our study confirms their findings even though we used different singleton and twin-specific birthweight charts. We also demonstrated that the association of HDP and PTB more closely resembles the association in singletons if the outcome (FGR or SGA) is assessed with a twin-specific chart. Moreover, we observed that the recently proposed sFGR diagnostic criteria applied to birthweight had a stronger and more consistent relationship with adverse events such as HDP and PTB. Couck et al. have studied the utility of the new Delphi consensus criteria for detecting perinatal adverse outcomes at different gestational ages and compared it to estimated fetal weight (EFW) discordance [10]. They reported similar predictive value but they used an EFW chart derived from a singleton population. Future studies could test the performance of twin-specific EFW standards in this context [27]. Furthermore, the utility and the choice of the various EFW and birthweight standards remain controversial.

### 4.4. Clinical and Research Implications

An initiative to facilitate and improve the quality of research in multiple pregnancy is ongoing [13,17]. The harmonization of the reported outcomes and their definitions is an important goal for reproducible research and robust summarization of evidence. However, harmonizing of the outcome definitions alone may not suffice as the reference standards also play a role in the assessment of outcomes such as fetal weight. Our findings suggest that twin-specific standards better reflect the association between HDP and adverse clinical outcomes such as FGR or SGA at birth. A perennial criticism of the twin-specific growth charts is that they may obscure pathological growth trajectories and under-detect at-risk fetuses. Our results show that birthweight anomalies, as assessed using twin-specific charts, have a better and more consistent association with adverse outcomes compared to assessment using singleton charts. Twin-specific charts were previously shown to reduce the rate of SGA diagnosis without increasing the rate of undetected SGA stillbirth cases [8]. Accumulating evidence suggests that twin-specific charts may be a viable option to reduce the over diagnosis of growth abnormalities and the resulting risk of iatrogenic prematurity, as well as better identification of the pregnancies at risk of complications such as HDP.

## 5. Conclusions

Fetal growth restriction in twin pregnancies has a stronger and more consistent association with adverse events such as HDP and PTB when using twin-specific rather than singleton charts. This study provides further evidence supporting the use of twin-specific charts when assessing fetal growth in twin pregnancies.

## Figures and Tables

**Table 1:** Characteristics of study cohort.

Variables	Singleton Pregnancies (*n* = 62,432)	Twin Pregnancies (*n* = 1473)	*p* *
Maternal age in years, median (IQR)	31.0 (27.0–35.0)	33.0 (30.0–36.0)	<0.001
Gestational age at birth in weeks, median (IQR)	40.0 (39.0–41.0)	36.9 (34.9–37.4)	<0.001
Smoker, *n* (%)	3673 (5.8)	75 (5.2)	0.350
Missing	0 (0.0)	47 (3.2)	
Self-reported ethnicity, *n* (%)			
Caucasian	32,264 (51.7)	826 (56.1)	<0.001
Afro-Caribbean	8996 (14.4)	216 (14.7)	0.812
Asian	8826 (14.1)	163 (11.1)	<0.001
Mixed	1535 (2.4)	15 (1.0)	<0.001
Other, prefer not to say	7587 (12.2)	150 (10.2)	0.0244
Missing	3224 (5.2)	103 (6.9)	
Chorionicity, *n* (%)			NA
DC	NA	1177 (80.0)	
MC	NA	296 (20.0)	
Hypertensive disorders of pregnancy, *n* (%)			
Gestational hypertension	1511 (2.4)	42 (2.9)	0.328
Preeclampsia	1590 (2.5)	110 (7.5)	<0.001
Birthweight in grams	3360 (3025–3700)	2430 (2058–2688) †	<0.001
Birthweight centile	42.8 (19.5–70.1)	40.1 (23.5–58.5) †	<0.001
Birthweight discordance %, median (IQR)	NA	9.4 (4.2–16.6)	NA

IQR: interquartile ranges, DC: dichorionic, MC: monochorionic, NA: not applicable. * Wilcoxon rank sum test or chi-square where appropriate. † Average birthweight for twins.

**Table 2:** The association of hypertensive disorders of pregnancy (HDP) and preterm birth (PTB) with small-for-gestational age (SGA) and fetal growth restriction (FGR) at birth in singleton pregnancies. The diagnostic criteria for SGA and FGR were <10th and <3rd weight centile, respectively. Selective fetal growth restriction (sFGR) for twins were diagnosed according to the Delphi consensus criteria.

Outcomes	Singleton SGA (Singleton Chart *)	*p* †	Twin SGA (Singleton Chart)	*p*	Twin SGA (Twin Chart ‡)	*p*
Delivery ≥34 weeks’ gestation without HDP	Reference	-	Reference	-	Reference	-
PTB <34 weeks’ gestation without HDP	1.43 (1.21–1.68)	<0.001	0.56 (0.42–0.74)	<0.001	3.10 (2.30–4.17)	<0.001
Delivery ≥34 weeks’ gestation with HDP	1.77 (1.61–1.94)	<0.001	1.41 (0.97–2.07)	0.074	2.12 (1.40–3.16)	<0.001
PTB <34 weeks’ gestation with HDP	10.26 (7.88–13.41)	<0.001	2.38 (1.08–5.79)	0.040	10.73 (4.83–26.22)	<0.001
**Outcomes**	**Singleton FGR** **(Singleton Chart)**	***p***	**Twin sFGR (Singleton Chart)**	***p***	**Twin sFGR (Twin Chart)**	***p***
Delivery ≥34 weeks’ gestation without HDP	Reference	-	Reference	-	Reference	-
PTB <34 weeks’ gestation without HDP	2.82 (2.28–3.44)	<0.001	1.03 (0.73–1.43)	0.872	3.55 (2.43–5.16)	<0.001
Delivery ≥34 weeks’ gestation with HDP	2.38 (2.07–2.71)	<0.001	1.36 (0.88–2.05)	0.160	2.17 (1.24–3.65)	0.004
PTB <34 weeks’ gestation with HDP	13.65 (10.36–17.86)	<0.001	2.41 (1.08–5.18)	0.025	5.69 (2.39–12.65)	<0.001

BW: birthweight, OR: odds ratio, CI: confidence interval, sFGR: selective fetal growth restriction, HDP: hypertensive disorders of pregnancy. * Birthweight was assessed using the standards from Poon et al. [23]. † Generalized linear model with logit link function. ‡ Birthweight was assessed using the standards from Ananth et al. [7].

**Table 3:** The association of hypertensive disorders of pregnancy (HDP) and preterm birth (PTB) with selective fetal growth restriction (sFGR) and small-for-gestational age in monochorionic and dichorionic twin pregnancies. The diagnostic criteria for SGA and FGR were <10th and <3rd weight centile, respectively. Selective fetal growth restriction was diagnosed according to the Delphi consensus criteria.

Outcomes	sFGR (Singleton Chart *)		sFGR (Twin Chart †)		SGA (Singleton Chart)		SGA (Twin Chart)	
	OR (95% CI)	*p* ‡	OR (95% CI)	*p*	OR (95% CI)	*p*	OR (95% CI)	*p*
*Dichorionic twin pregnancies*								
Delivery ≥34 weeks’ gestation without HDP	Reference	-	Reference	-	Reference	-	Reference	-
PTB <34 weeks’ gestation without HDP	0.71 (0.45–1.09)	0.126	2.42 (1.50–3.80)	<0.001	0.46 (0.32–0.64)	<0.001	2.62 (1.84–3.72)	<0.001
Delivery ≥34 weeks’ gestation with HDP	1.49 (0.93–2.32)	0.084	2.01 (1.09–3.53)	0.018	1.37 (0.91–2.07)	0.135	1.91 (1.22–2.95)	0.003
PTB <34 weeks’ gestation with HDP	2.23 (0.92–5.10)	0.061	4.62 (1.74–11.08)	0.001	2.85 (1.18–7.91)	0.028	11.98 (4.94–33.45)	<0.001
*Monochorionic twin pregnancies*								
Delivery ≥34 weeks’ gestation without HDP	Reference	-	Reference	-	Reference	-	Reference	-
PTB <34 weeks’ gestation without HDP	2.01 (1.11–3.63)	0.020	9.19 (4.32–20.7)	<0.001	0.87 (0.50–1.49)	0.608	5.53 (3.02–10.27)	<0.001
Delivery ≥34 weeks’ gestation with HDP	0.71 (0.16–2.26)	0.599	3.24 (0.68–11.7)	0.093	1.66 (0.64–4.63)	0.305	3.60 (1.25–9.78)	0.013
PTB <34 weeks’ gestation with HDP	3.78 (0.44–32.31)	0.189	17.27 (1.93–155.7)	0.006	0.97 (0.11–8.21)	0.976	6.18 (0.72–53.22)	0.074

sFGR: selective fetal growth restriction, HDP: hypertensive disorders of pregnancy, BW: birthweight, OR: odds ratio, CI: confidence interval. * Generalized linear model with logit link function. † Birthweight was assessed using the standards from Poon et al. [23]. ‡ Birthweight was assessed using the standards from Ananth et al. [7].

**Table 4:** The area under the curve (AUC) values of the various study outcomes predicted by gestational age at delivery and the occurrence of hypertensive disorders of pregnancy. The diagnostic criteria for SGA and FGR were <10th and <3rd weight centile, respectively. Selective fetal growth restriction was diagnosed according to the Delphi consensus criteria.

Outcome	AUC (95% CI), Singleton Standard *	AUC (95% CI), Twin Standard †	*p* ‡
All twin pregnancies			
sFGR at birth	0.52 (0.49–0.55)	0.64 (0.60–0.68)	< 0.001
Any SGA at birth	0.56 (0.54–0.58)	0.62 (0.59–0.65)	< 0.001
Dichorionic twin pregnancies			
sFGR at birth	0.54 (0.51–0.58)	0.60 (0.55–0.65)	0.028
Any SGA at birth	0.57 (0.54–0.59)	0.61 (0.58–0.64)	0.026
Monochorionic twin pregnancies			
sFGR at birth	0.58 (0.52–0.65)	0.75 (0.68–0.83)	< 0.001
Any SGA at birth	0.53 (0.47–0.58)	0.69 (0.63–0.76)	< 0.001

sFGR: selective fetal growth restriction, SGA: small-for-gestational-age, AUC: area under the curve, CI: confidence interval. * Birthweight was assessed using the standards from Poon et al. [23]. † Birthweight was assessed using the standards from Ananth et al. [7]. ‡ De Long’s test, one-tailed.

## Supplementary Materials

The following are available online at https://www.mdpi.com/2077-0383/9/8/2518/s1, Figure S1: The receiver-operating characteristics curves of selective fetal growth restriction (sFGR) (a) and small-for-gestational age (SGA) at birth (b) in twin pregnancies as assessed using singleton and twin standards. The areas under the curve were significantly higher when the outcomes were assessed with twin standards (*p* < 0.05 for all). The difference persisted in the subgroup analyses according to chorionicity.
