# Supplementary material for: The Association Between Hypertension in Pregnancy and Preterm Birth with Fetal Growth Restriction in Singleton and Twin Pregnancy: Use of Twin Versus Singleton Charts
Source: J Clin Med. 2020 Aug 5;9(8):2518. doi: 10.3390/jcm9082518 (PMC7464003; doi:10.3390/jcm9082518)
Supplement: Supplementary file 1 [file jcm-09-02518-s001.pdf]

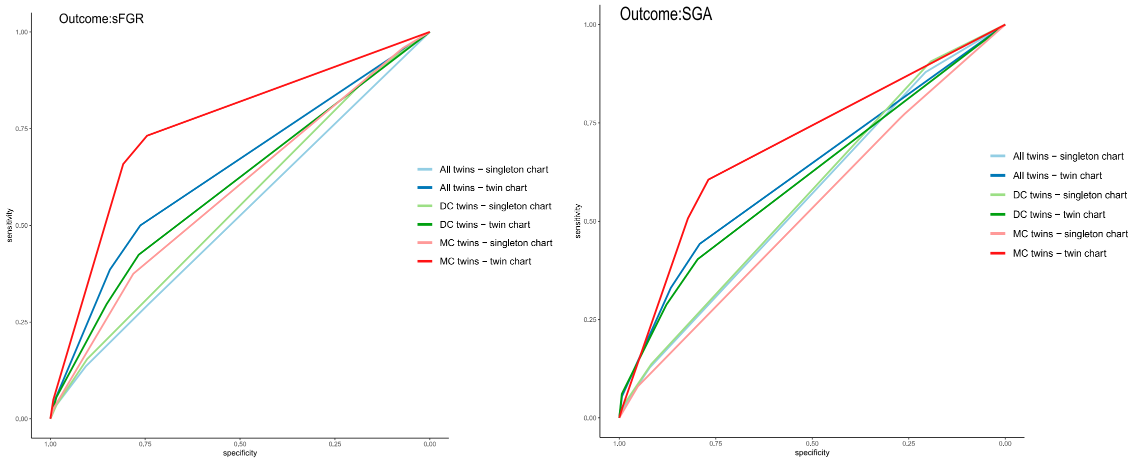

Figure S1. The receiver operating characteristics curves of selective fetal growth restriction (sFGR) (a) and small-for-gestational age (SGA) at birth (b) in twin pregnancies as assessed using singleton and twin standards. The area under the curve were significantly higher when the outcomes are assessed with twin standards ($p<0.05$ for all). The difference persisted in the subgroup analyses according to chorionicity.

MC: monochorionic, DC: dichorionic
